# Interferon regulatory factor 1: a key transcriptional node orchestrating macrophage polarization: mechanisms and therapeutic potential

**DOI:** 10.3389/fimmu.2026.1884705

**Published:** 2026-10-21

**Authors:** Shuqi Cao, Shuang Feng, Yue Gao, Zhiyuan Sun, Shiyao Bai, Xinming Su

**Affiliations:** Department of Respiratory and Critical Care Medicine, The First Hospital of China Medical University, Shenyang, China

**Keywords:** epigenetic regulation, immunometabolism, interferon regulatory factor 1, macrophage polarization, non-coding RNAs, tumor microenvironment

## Abstract

Macrophageexhibit remarkable functional plasticity and adopt M1-like pro-inflammatory or M2-like anti-inflammatory/pro-resolving states in response to microenvironmental stimuli. Notably, this binary functional classification originates largely from *in-vitro* experimental systems; tissue-resident macrophages *in vivo* display a continuous spectrum of activation phenotypes rather than discrete subsets. Dysregulated macrophage polarization drives the pathogenesis of infectious diseases, malignancies, and metabolic-inflammatory disorders. Interferon regulatory factor 1 (IRF1) is a pivotal transcription factor governing macrophage fate, yet its multilayered regulatory circuits and context-dependent dual impacts on M1-like and M2-like transcriptional programs remain to be systematically summarized. This review synthesizes recent advances in IRF1-centered regulatory networks of macrophage polarization. We recapitulate IRF1 crosstalk with core signaling cascades including JAK/STAT, TLR/MyD88 and IFN-γ pathways, as well as its transcriptional control of target genes such as iNOS, IL-12, and CXCL10. Multi-layer regulatory mechanisms modulating IRF1 activity are outlined, including transcriptional, epigenetic, non-coding-RNA-mediated post-transcriptional, and post-translational modifications. We further dissect IRF1-linked immunometabolic reprogramming and epigenetic remodeling, and describe its versatile pathophysiological roles in infection, cancer and metabolic inflammatory diseases based on multi-omics and single-cell sequencing evidence. Collectively, this review illustrates IRF1 as a key transcriptional node that orchestrates macrophage polarization, and highlights prospective therapeutic targets and intervention strategies for IRF1-associated immune-mediated disorders.

## 1 Introduction

Macrophages are highly plastic innate immune cells that dynamically remodel their functional profiles in response to microenvironmental cues, adopting pro-inflammatory M1-like or anti-inflammatory and pro-resolving M2-like states (1, 2). The traditional M1/M2 dichotomy, while foundational, is increasingly recognized as an oversimplification of the continuous activation states seen *in vivo*, a concept supported by broader reviews of macrophage biology (1). A balanced polarization state is essential for maintaining immune homeostasis, facilitating effective pathogen clearance, and supporting tissue repair processes (3). During inflammation, macrophages initially adopt an M1 phenotype to eliminate threats, but must subsequently transition toward an M2 phenotype to resolve inflammation and promote regeneration, thereby preventing chronic tissue damage (2). Conversely, dysregulated macrophage polarization contributes significantly to the pathogenesis of various diseases, including infectious disorders, cancer, metabolic syndromes, and autoimmune conditions (4, 5). Decoding the molecular circuits governing macrophage fate, such as those involving NF-κB, STAT, and PPARγ signaling, is therefore critical for developing innovative immunomodulatory therapeutic strategies (6). Targeting these polarization mechanisms offers promising avenues for treating inflammatory and immune-mediated diseases, although further research is needed to translate these insights into precise clinical interventions (7).

Interferon regulatory factors (IRFs) constitute a pivotal family of transcription factors that orchestrate innate immune responses by integrating signals from pattern recognition receptors and cytokines (8). Within this family, IRF4 has been characterized as a regulator facilitating M2-like programming, while other members such as IRF5 are known to drive pro-inflammatory outputs (9). Distinct from these homologs, IRF1 functions as an early-response integrator that converges interferon signaling, pattern-recognition receptor inputs, and metabolic stimuli to shape macrophage identity (10, 11). Loss-of-function models suggest that IRF1 deficiency impairs M1-associated transcriptional programs and may enhance alternative activation markers (10, 11). These functional data support the conceptualization of IRF1 as a molecular switch governing macrophage fate decisions, although its biological output is not an absolute digital toggle but rather heavily dependent on cell type and microenvironmental context. Consequently, IRF1 exhibits dual, context-dependent roles across diverse pathological settings, including infection, tumor immunity, and sterile inflammatory injury, where it may exert either protective or detrimental effects depending on the specific immunological milieu (12). The activity of IRF1 is fine-tuned via multilayered regulatory circuits encompassing transcriptional, epigenetic, post-transcriptional, and post-translational mechanisms, which collectively determine its stability and transcriptional potency (13).

In this review, we synthesize recent advances concerning IRF1-centered regulatory networks in macrophage polarization. We first describe IRF1 structural features and its multi-layered regulatory mechanisms, followed by mechanistic dissection of how IRF1 governs M1-like programming and restrains M2-like phenotypes. We further discuss the crosstalk among IRF1-driven immunometabolic reprogramming, epigenetic remodeling and non-coding-RNA-mediated regulation. We summarize the pathophysiological roles of IRF1 in multiple disease conditions, evaluate current therapeutic strategies targeting IRF1 signaling, and highlight existing knowledge gaps and future research perspectives. This work aims to provide an integrated understanding of IRF1-mediated macrophage fate control and offer insights for developing context-specific therapeutic interventions for IRF1-related human diseases.

## 2 Biological characteristics of IRF1 and its expression regulation in macrophages

### 2.1 Structure and basic functions of IRF1

The IRF family comprises evolutionarily conserved transcription factors that orchestrate innate immune responses across vertebrates, with phylogenetic analyses indicating their origin coincides with the emergence of multicellularity (13). While individual members exhibit distinct functional specializations, such as IRF3 driving type-I interferon responses upon viral pattern recognition and IRF4 and IRF5 governing M2-like and M1-like macrophage polarization respectively, these roles are integrated within a broader network of immune regulation (14–17). Furthermore, IRF2 functions predominantly as a transcriptional repressor by competing for DNA-binding sites, thereby fine-tuning the magnitude and duration of interferon-induced antiviral gene expression (18).

Within this diverse family, IRF1 is an important transcription factor that has a highly conserved N-terminal domain (DNA-binding domain or DBD), which is responsible for binding directly to target genes’ promoter regions, and a C-terminal regulatory domain (also known as transactivation domain). The DBD is a helix-turn-helix structure that specifically binds to specific DNA sequences called ISREs and IRF-binding elements (IRF-Es) in response to the action of interferons to stimulate transcription (19–21). This particular arrangement of IRF1 appears to have been evolutionarily conserved throughout the history of vertebrates and can be found in many different vertebrate species (i.e., amphibians, fish, and mammals), some with additional functional protein domains, such as an IRF-association domain (IAD), and the presence of nuclear localization signals (NLS) (21, 22).

IRF1 is constitutively expressed at low basal levels but can be rapidly and robustly induced upon exposure to diverse stimuli, including IFN-γ, various cytokines, and pathogen-associated molecular patterns (PAMPs) such as LPS or poly(I:C) (19, 20, 23, 24). For example, the human metapneumovirus uses the IRF1 gene via TBK1 and type I IFN (IFN-β) to create a positive feedback loop for enhancing the immune response (23). Therefore, IRF1 is a major link between PRRs and IFN receptors; it transduces signals from TLRs and other PRRs to activate ISGs (24–26). In addition to well-established antiviral activity, IRF1 also enhances antibacterial host defense; for example, IRF1 overexpression in fish increases bacterial resistance and enhances the effectiveness of vaccine adjuvants (21).

In terms of its mode of action, IRF1 functions primarily as an activator of transcription but can also function as a repressor depending on the specific circumstances. The response to interferon is finely tuned by competition between IRF1 and other members of the IRF family (e.g., IRF2 and IRF10) for ISRE binding sites (22, 27). IRF1’s overall activity is controlled through transcriptional regulation, protein stability, and post-translational modifications (e.g., phosphorylation and acetylation of its NLS), all of which impact nuclear localization and transcriptional activity (28, 29). In structural cells, mitochondrial DNA leakage induced by ionizing radiation results in IRF1 nuclear localization via STING/p300-mediated post-translational modifications. Furthermore, IRF1 stability is maintained through ubiquitination (e.g., via TLR25/MyD88 and K63-linked ubiquitination in teleosts), which contributes to the stabilization of IRF1 and promotes the expression of genes encoding interferon (28). Furthermore, IRF1 stability is regulated by ubiquitination; in teleosts, TLR25/MyD88 signaling promotes K63-linked ubiquitination to stabilize IRF1 and upregulate interferon gene expression (29). This complex and highly regulated process enables IRF1 to quickly initiate and accurately terminate the immune response and plays a significant role in defining the outcome of infection, inflammatory reactions, and/or cancer as a master regulator of the innate immune system (24, 30).As shown in **Figure 1**, IRF1 contains four conserved functional domains that collectively determine its transcriptional activity and regulatory versatility.

**Figure 1:**
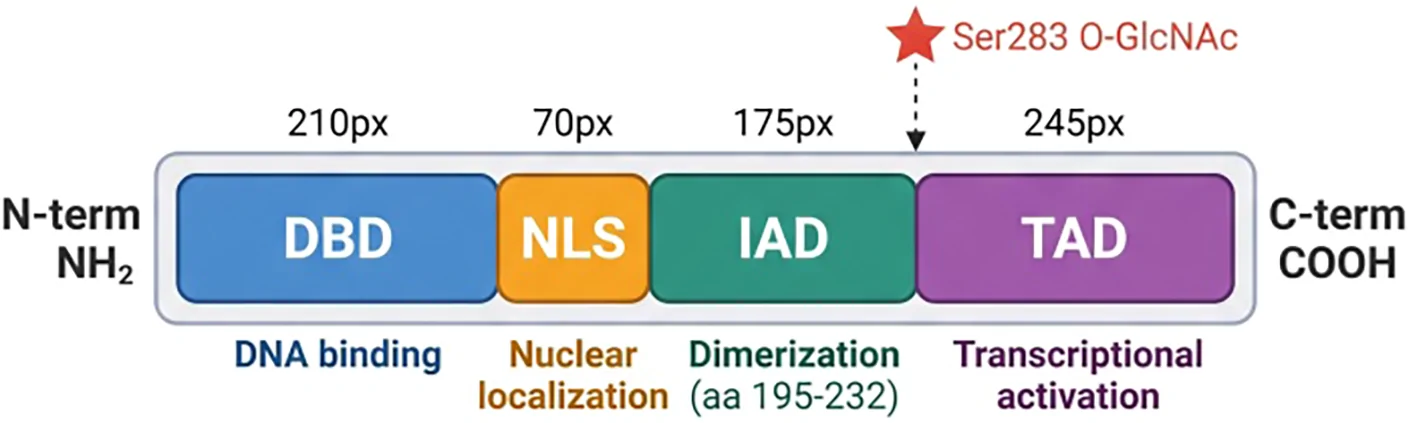
Schematic diagram of IRF1 protein domain structure.

### 2.2 Transcriptional and post-transcriptional regulation of IRF1 expression in macrophages

IRF1 is primarily activated via the JAK1/JAK2-STAT1 signaling system by non-canonical (outside of direct IFN-γ stimulation) TLR4 (and MyD88) pathways (via the binding of LPS) and can also be upregulated by TRIF pathway-dependent signaling (via the activation of TBK1/IKKϵ and IRF3) (31). During TLR4-mediated signaling, the IRF1 promoter contains an FXRE (TTCN3) box with a high affinity for a phosphorylated dimer of STAT1, allowing for increased transcription of both whi1 and Irf1 (31). STAT1, once phosphorylated and then dimerized, will translocate into the nucleus upon IFN-γ activation. The transcription of IRF1 from the IRF1 promoter is induced robustly by IFN-γ and is critical for the development of anti-mycobacterial immunity (32). Finally, during viral infection such as the HMPV (Human Metapneumovirus), IRF3 and IRF1 are also equally important, as TRIF dependent signaling activates TBK1/IKKϵ-IRF3 and the induction of IRF1 (23, 32).

The activation of optimal macrophage responses requires coordinating multiple signaling pathways to allow for epigenetic reprogramming. For instance, the formation of new enhancers in response to LPS requires both NF-κB and ISGF3 (which is activated through the IRF3/type I IFN pathway) (33). In a similar manner, the action of IFN-γ to form STAT1 homodimers (GAF) in combination with IRF1 opens chromatin that would not normally be accessible and facilitates the creation of new enhancers (33). Other transcription factors are also involved in modulating this regulatory network: the master regulator SUB1 (PC4) promotes the transcription of Irf1 and enhances M1 polarization in atherosclerosis through a CK2-dependent mechanism (34), while CITED2 acts as an intrinsic negative regulator by inhibiting the STAT1-IRF1 signaling axis (35). Additionally, Zhx2 is associated with NF-κB p65 and directly interacts with the Irf1 promoter, promoting macrophage anti-tumor responses (36).

The regulation of IRF1 transcription is determined by epigenetics. IRF1 expression is affected by decreased H3K4me3 enrichment at its promoter due to methionine shortage, which leads to inadequate macrophage polarization (37). In contrast, H3K9me2 demethylation at the Irf1 promoter occurs when STAT1 and JMJD1A (KDM3A) work together to increase IRF1 expression, allowing our bodies to fight off intestinal bacterial infections (38). In osteoarthritis, GCN5 and SETD2 are recruited by IRF1 to modify chromatin near target genes such as SMARCC1, thereby influencing the skewing of macrophages (39).

MicroRNAs (miRNAs) and long non-coding RNAs (lncRNAs) regulate the levels of IRF1 post-transcriptionally; this regulation often occurs via feedback circuits. For instance, the miRNAs miR-130b-3p and miR-19a-3p are downregulated in M1 polarization, where miR-130b-3p targets the 3’-UTR of Irf1 mRNA and miR-19a-3p targets STAT1 to inhibit IRF1 expression indirectly (40, 41). Conversely, the miRNAs miR-106b-5p and exosomal miR-21 enhance M2 polarization by inhibiting the IRF1 axis in glioblastoma and lung cancer, respectively (42–44). Nrf2-induced miR-93-5p and AhR-induced miR-142a are examples of miRNAs that inhibit M1 polarization through targeting of Irf1 mRNA in calcium oxalate nephrocalcinosis (45, 46). In the context of lncRNAs, the lncRNA CARINH controls the expression of its complementary gene, IRF1, to coordinate antiviral responses (47, 48), while IRF1-AS1 is co-regulated with IRF1 in macrophages infected by Leishmania, suggesting a cis-regulatory mechanism (49).

Post-translational modifications can play a role to fine tune the activity of IRF1 (50, 51). For instance, O-GlcNAcylation at Ser283 by OGT was shown to regulate the polarization of macrophages in heart failure and IRF1 is also involved in amplifying M1 polarization during septic ARDS by binding to the Stat1 promoter to form a positive feedback loop. In addition (52). IRF1 activates genes like SLC7A11 which are involved in regulating ferroptosis in liver fibrosis and enhances transcription of IGFBP7 (53), which has an influence on macrophage pyroptosis during kidney injury (54). These different levels of regulation, which can be found in **Table 1**, are critical to determining the fate and function of macrophages during different physiological and pathological conditions.

**Table 1:** Molecules that regulate IRF1 expression and activity.

IRF1-activating molecules			
Transcriptional (promoter binding/co-activation)			
**Molecule**	**Mechanism**	**Disease/Model**	**References**
STAT1	Binds GAS element in *IRF1* promoter	IFN-γ stimulation, mycobacterial infection	(31, 33)
NF-κB (p65)	Cooperates with Zhx2 to bind *IRF1* promoter	LPS, tumor microenvironment	(36)
SUB1 (PC4)	CK2-dependent activation of *Irf1* transcription	Atherosclerosis	(34)
Zhx2	Interacts with *IRF1* promoter (with p65)	Macrophage anti-tumor responses	(36)
Epigenetic (activating marks/demethylation)			
**Molecule**	**Mechanism**	**Disease/Model**	**References**
H3K4me3	Enrichment at *IRF1* promoter (promotes expression)	Renal ischemia-reperfusion (methionine-sensitive)	(37)
JMJD1A (KDM3A)	Demethylates H3K9me2 at *IRF1* promoter	Enteric bacterial infection	(38)
Post-transcriptional (lncRNA-mediated)			
**Molecule**	**Mechanism**	**Disease/Model**	**References**
CARINH (lncRNA)	Regulates expression of antisense gene *IRF1*	Viral infection	(48)
IRF1-AS1	Co-regulated with *IRF1* (cis-regulatory)	Leishmania infection, IBD	(49, 55)
Post-translational (stabilization/activating PTMs)			
**Molecule**	**Mechanism**	**Disease/Model**	**References**
OGT	O-GlcNAcylation at Ser283	Heart failure	(51)
p300/STING	Acetylation/phosphorylation regulate nuclear translocation	Radiation-induced inflammation	(28)
CK2	Modulates IRF1 chromatin binding (phosphorylation)	Antiviral signaling	(56)
**IRF1-inhibiting molecules**			
Transcriptional (repression)			
**Molecule**	**Mechanism**	**Disease/Model**	**References**
CITED2	Restrains STAT1-IRF1 signaling axis	Atherosclerosis	(35)
Epigenetic (repressive/loss of activating marks)			
**Molecule**	**Mechanism**	**Disease/Model**	**References**
H3K4me3 (reduced)	Methionine restriction reduces enrichment → IRF1c	Renal ischemia-reperfusion	(37)
Post-transcriptional (miRNA-mediated)			
**Molecule**	**Mechanism**	**Disease/Model**	**References**
miR-130b-3p	Targets *IRF1* mRNA 3R-UTR	M1 polarization, acute lung injury	(40)
miR-19a-3p	Targets *STAT1* (indirectly inhibits IRF1)	M1 polarization	(41)
miR-106b-5p	Targets IRF1, suppresses IFN-β signaling	Glioblastoma M2 polarization	(42)
miR-103	Targets *STAT1*, suppresses STAT1/IRF1 axis	Recurrent spontaneous abortion	(43)
miR-21	Tumor-derived exosomal miR-21 targets IRF1	Lung cancer M2 polarization	(44)
miR-93-5p	Nrf2-activated, targets *TLR4* and *IRF1*	Calcium oxalate nephrocalcinosis	(45)
miR-142a	AhR-activated, targets *IRF1* and *HIF-1α*	Calcium oxalate nephrocalcinosis	(46)
miR-23a-3p	BMSC-exosome-delivered, targets IRF1	Tendon-bone healing	(57)
miR-24-3p	MSC-EV-delivered, targets IRF1	Inflammatory bowel disease	(58)
Post-translational (ubiquitination/degradation)			
**Molecule**	**Mechanism**	**Disease/Model**	**References**
TRIM21 (E3)	Promotes K48-linked ubiquitination of IRF1	Sepsis-induced acute lung injury	(59)
SPOP (E3)	Targets IRF1 for proteasomal degradation	Basic research	(60)

BMSC, bone marrow stromal cell; EV, extracellular vesicle; IBD, inflammatory bowel disease; PTM, post-translational modification.3 IRF1 up-regulation drives M1-type macrophage polarization and inflammatory responses.

Bold text denotes column headings and category labels.

### 3.1 Direct transcriptional activation of M1 signature genes

Interferon regulatory factor 1 (IRF1) functions as a master transcriptional regulator that drives the differentiation and polarization of macrophages toward the pro-inflammatory M1 phenotype, thereby orchestrating innate immune responses (15, 17). A central mechanism underlying this polarization involves the direct binding of IRF1 to the promoter region of inducible nitric oxide synthase (iNOS), which initiates the transcription of iNOS and subsequent production of nitric oxide (NO) (18). This NO-mediated pathway is critical for the antimicrobial and antitumor effector functions characteristic of classically activated macrophages (61). Experimental evidence demonstrates that silencing IRF1 using siRNA in bone marrow-derived macrophages significantly attenuates iNOS expression, confirming the dependency of this marker on IRF1 activity. Furthermore, during ischemia-reperfusion of the liver, IRF1 activation is contingent upon the iNOS/NO signaling axis, establishing a positive feedback loop that amplifies inflammatory tissue damage (62). Beyond iNOS regulation, IRF1 collaborates with other transcription factors, such as NF-κB, to synchronize the expression of key pro-inflammatory cytokines including IL-12 subunits and TNF-α, which collectively promote Th1-type immunity (63).

In addition to being able to induce the production of cytokines, IRF1 can also induce specific subclasses of chemokines, which are involved in the recruitment of effector immune cells to sites of inflammation and/or tumor development. Specifically, IRF1 can stimulate the production of three chemokines - CXCL9, CXCL10 and CXCL11 - that are capable of serving as ligands for the CXCR3 receptor on Th1 cells and cytotoxic T lymphocytes (64). As demonstrated by the nearly abolishment of CXCL10 expression in siIrF1-BMDMs compared to wild-type cells, induction of CXCL10 is a highly sensitive, and specific outcome of IRF1 activation (64). Notably, the IRF1-CXCL10 axis has major clinical implications in oncology; for example, in ovarian cancer, tumors that are characterized by extensive immune infiltration (through the presence of M1 types of tumor-associated macrophages (TAMs) near T cells) are often associated with enhanced IRF1 activity (65). In colorectal cancer, the IRF1-mediated upregulation of CXCL10 promotes CD8+ T cell infiltration and reduces tumor growth, supporting the role of IRF1 in effective anti-tumor immunity (66).

The IRF1 gene is an essential part of many different pathologies, such as acute respiratory distress syndrome (ARDS) and recurrent spontaneous abortion (RSA), and contributes to the pathologic M1 polarization through activation of the STAT1/IRF1 pathway (43). In contrast, IRF1 is down-regulated to suppress M1 polarization. This occurs when the aryl hydrocarbon receptor (AhR) is activated, which protects calcium nephrocalcinosis from M1 polarized macrophage activation through the induction of miR-142a, which is a direct inhibitor of IRF1 and HIF-1α (46). Similarly, in glioblastoma, miR-106b-5p shifts macrophage polarization to an M2 phenotype through down-regulation of the IRF1/IFN-β pathway (42). Together, this data suggests that the ability of IRF1 to regulate iNOS, pro-inflammatory cytokines, and chemokines by direct transcriptional control provides the molecular basis for the differentiation of M1 macrophages.

### 3.2 Amplification and synergy with STAT1 signaling

There is a large degree of functional similarity between the transcriptive factors IRF1 and STAT1, which work together to promote macrophage M1 polarization generated through synergistic interactions. This mutual reinforcement occurs in that; through the transactivation of common target genes STAT1 promotes and enhances the transactivation of binding sites on IRF1 (41). The activation of IRF1 by STAT1 will further augment the expression and activity of IRF1. Based upon their established synergy, any interruption to the STAT1/IRF1 axis, by means of pharmacological agents such as the flavonoid fisetin or the specific inhibitor fludarabine, will consistently inhibit the expression of the typical M1 polarization markers, suggesting that these factors cooperate in inducing systemic inflammation (67, 68). This relationship between IRF1 and STAT1 has also been identified in the pathogenesis of atherosclerosis, whereby co-activation of the two transcription factors results in enhanced expression of pro-inflammatory genes which are inhibited by CITED2 thereby limiting plaque development (35). In cancer immunotherapy, there are strategies (e.g. ARTESUNATE) that utilize the synergistic interaction of IRF1 and STAT1 to stabilize the M1 anti-tumor phenotype of tumor-associated macrophages (TAM) following drug treatment (69). Furthermore, through complex feed-forward loops involving IRF1/poly(ADP-ribose)polymerase 1(PARP1 )and IRF1/STAT1 a robust inflammatory transcriptional network is created that stabilizes the inflammatory state of macrophages.

The relationship between the two arms of this synergy is primarily based on a strong positive feedback mechanism involving interferon-stimulated genes (ISGs) by way of a transcription factor. As an exemplar of this relationship, the transcription factor IRF1 is induced through IFN-γ-mediated activation of STAT1 (70). Once IRF1 is expressed, it will act as a transcription factor to increase the expression of multiple additional ISGs and will also help to maintain the M1 phenotype (52). The synergy between these two arms of the immune system as depicted in a septic injury model of lung damage demonstrates that IFIH1-activated IRF1 promotes the production of inflammatory mediators. In addition, similar mechanisms of cross-regulation among the three components of the DcR3/STAT1/IRF1 feedback loop are also observed in the pathogenesis of pancreatic cancer; in the context of this disease, DcR3 activates both STAT1 and IRF1, and once activated, IRF1 promotes the expression of DcR3, thus perpetuating a cycle of tumor development (71). This feedback loop provides a mechanism for a rapid and prolonged inflammatory response, causing macrophages to be stuck in an M1 state (i.e. to be unable to change to an M2 phenotype) and therefore, blocking the phosphorylation of STAT1 leads to downregulation of both IRF1 and downstream ISGs (67).

The IRF1-STAT1 axis is crucial for mounting an adaptive response to intracellular pathogens and developing anti-tumor immune responses. The histone demethylase JMJD1A partners with STAT1 to trigger the transcription factor IRF1 during the course of infection with Citrobacter rodentium to promote expression of the chemokine CCL8 and recruit protective immune cells (38). Similarly, in the setting of viral infection with human metapneumovirus, convergent activation of TBK1, IFN-β and IRF3 pathways results in STAT1-mediated upregulation of IRF1, which is required for the antiviral response (23). In the field of oncology, the IRF1-STAT1 axis determines the anti-tumor activity of the immune microenvironment. For example, in ovarian cancer, the presence of CXCL10 secreting, IRF1+ and pSTAT1+ M1 TAMs correlates with robust T-cell activation and improved clinical outcome (65). In contrast, the activation status of the IFN-γ/p-STAT1/IRF1 pathway of tumor-infiltrating NK cells in patients with hepatocellular carcinoma (HCC) is associated with immune evasion and restoration of this axis is required to recover anti-tumor activity (72). Nonetheless, this axis also affects essential immune checkpoints such as IFN-γ-induced PD-L1 expression via a JAK1/STAT1/IRF1 signaling pathway whereby tumors hijack this pathway for escaping from the immune response (73). Therefore, the cooperation between IRF1 and STAT1 represents both a critical component for the defense of the host but is also subject to pathological alteration.

The overall molecular events that are involved in IRF1-mediated M1 polarization (including the upstream integration of signals, activation of downstream targets and creating a positive feedback loop with STAT1) are presented in **Figure 2**.

**Figure 2:**
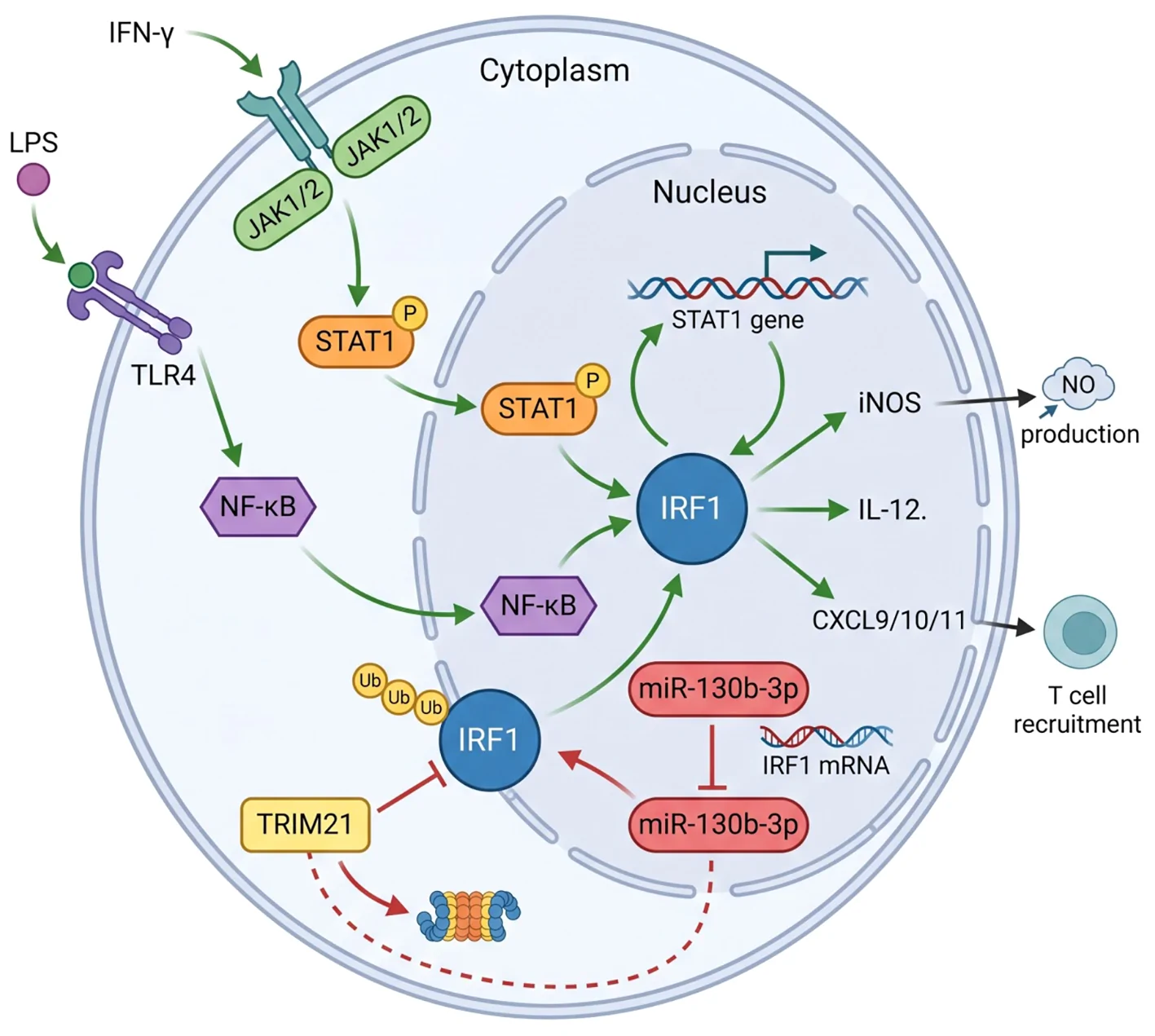
Core molecular mechanisms by which IRF1 drives M1 macrophage polarization.

This schematic illustrates the primary signaling networks governing IRF1-mediated classical (M1) macrophage polarization. Left panel (Upstream activation): IFN-γ activates the JAK1/2-STAT1 pathway, where STAT1 dimers bind to GAS elements in the Irf1 promoter to induce its transcription. LPS triggers TLR4 signaling via both MyD88-dependent and TRIF-dependent pathways to activate NF-κB and IRF3, culminating in IRF1 expression. TBK1 and IFIH1 also serve as crucial upstream regulators. Center panel (IRF1): IRF1 functions as a transcription factor comprising a highly conserved DNA-binding domain (DBD), an IRF-association domain (IAD), and nuclear localization signals (NLSs). Right panel (Downstream target genes): IRF1 directly transactivates M1 signature genes, including iNOS (driving NO production), IL-12, TNF-α, and specific chemokines (CXCL9, CXCL10, and CXCL11). Moreover, IRF1 promotes STAT1 transcription, establishing a robust positive feedback loop. Bottom panel (Negative regulation): Specific miRNAs (including miR-130b-3p and miR-19a-3p) bind to the Irf1 3′-UTR to repress its expression. Additionally, IRF1-induced SOCS1 exerts negative feedback on JAK-STAT signaling, while E3 ubiquitin ligases (e.g., TRIM21) mediate the K48-linked ubiquitination and subsequent proteasomal degradation of the IRF1 protein. Arrows indicate activation or promotion; T-bars represent inhibition.

## 4 IRF1 down-regulation de-represses M2-type macrophage programming

### 4.1 Suppressing the expression of M2 marker genes

IRF1 functions as a pivotal transcriptional repressor of alternatively activated (M2) macrophage polarization, thereby antagonizing the M2 program through both direct and indirect regulatory mechanisms (15). This suppression is critical for maintaining immune homeostasis, as IRF1 helps commit monocytes to the pro-inflammatory M1 phenotype while restricting anti-inflammatory M2 differentiation. A major indirect mechanism involves the competition for critical transcriptional co-activators, specifically SUB1 (PC4), which has been identified as a master regulator of the Toll-like receptor (TLR) response in atherosclerosis (34). SUB1-driven induction of IRF1 transcription is dependent on casein kinase 2 (CK2), and Sub1-knockout macrophages exhibit significantly reduced Irf1 expression, leading to attenuated M1 polarization and enhanced M2 skewing (34). The relationship between macrophage polarization states and plaque stability is multifaceted, with evidence supporting roles for both M1 and M2 phenotypes in different stages of atherosclerosis (74, 75).

Moreover, IRF1 is part of a regulatory circuit where inhibition of IRF1 must occur prior to the expression of M2 genes. In colorectal tumors, radio-resistant cells overexpress miR-1226-5p which downregulates IRF1. This downregulation, through the transfer of miR-1226-5p from the tumor cells to the macrophages, activates M2 differentiation in the macrophages, as evidenced by the upregulation of specific M2 markers (76). Similarly, within the glioblastoma microenvironment, miR-106b-5p is upregulated in TAMs and directly inhibit IRF1/IFN-β signaling pathway, facilitating their M2 differentiation (42) from multiple lines of evidence support the conclusion that the downregulation of IRF1, primarily via miRNA targeting, releases this inhibition on M2 differentiation programs.

The role of IRF1 as an antagonist to M2 marker expression has been validated in a variety of biological systems and contexts. For example, in microglial cells (resident macrophages of the CNS), IRF1-knockout (IRF1-KO) microglia show a decrease in proinflammatory M1 marker levels after LPS stimulation and a corresponding increase in IL-4-/IL-13-driven expression of anti-inflammatory M2 markers (e.g., Arg-1, CD206, and TGF-β) (77). As such, these results demonstrate that IRF1 plays a critical role in the skewing of the polarization balance away from the IL-4-/IL-13-mediated M2 state. Additionally, multi-omics sequencing of bone marrow-derived macrophages (BMDM) has shown that IRF1 is the most significantly upregulated transcription factor in M1 macrophages, and knockdown of IRF1 results in a significant reduction in IFN-γ-mediated gene expression, thereby confirming that IRF1’s regulatory network drives inflammation in a manner that is functionally antagonistic to M2 polarization (64).

Conversely, the overexpression or activation of IRF1 consistently exerts significant inhibition on IL-4/IL-13-induced M2 marker expression. Bioinformatics and experimental validation revealed that miR-23a-3p, which is highly enriched in these exosomes, directly targets IRF1. The overexpression of miR-23a-3p in BMSC-Exos robustly enhanced M2 polarization, functionally confirming that the suppression of IRF1 facilitates the expression of M2-associated genes (57). Interventions enhancing IRF1 activity or stability repress M2-like markers. In HCC, non-SUMOylated nuclear IL-33 triggers ubiquitin-dependent IRF1 degradation and lowers PD-L1 transcription. IL-33 SUMOylation abolishes this interaction to stabilize IRF1 but paradoxically promotes M2-like polarization, demonstrating dynamic control of M2 phenotype by IRF1 protein stability (78). In inflammatory bowel disease, small extracellular vesicles (sEVs) derived from TNF-α-pretreated menstrual blood-derived mesenchymal stem cells effectively alleviated colitis. This therapeutic effect was accompanied by M2 macrophage polarization, mediated by upregulated miR-24-3p within the sEVs targeting and downregulating IRF1 expression in the colon (58).

IRF1 has a strong and significant effect on the IL-4/IL-13 signaling pathway, mainly because it works against the actions of the primary transcription factor for M2 polarization (STAT6). In allergic asthma, a new pathogenic subset of IL-9+ macrophages has been defined: M(IL-33+IL-2). The polarization of these macrophages requires the activation of the JAK2-STAT3-IRF1 pathway, which indicates that IRF1 may function downstream of cytokine signals other than the traditional IL-4/STAT6 axis in allergic asthma, and it may be competing with the traditional IL-4/STAT6 axis (79). Furthermore, IRF1-driven M1-like gene transactivation underpins its transcriptional antagonism to M2-like polarization. In Leishmania donovani infection, infection-induced M2-promoting miR-146a-5p is neutralized, lowering M2 markers and p-STAT6 and increasing M1-inducing transcription factors such as IRF1 (80). This supports the idea that IRF1 and STAT6 bring about reciprocal inhibition of their respective transcriptional programs.

On the other hand, when comparing spinal cord injury models, the expression of heme oxygenase-1 (HO-1) in microglia was associated with increasing M2 polarization while decreasing neuroinflammation. The mechanism by which HO-1 exerted this cytoprotective effect involved HO-1 inducing the IRF1/DRP1 pathway to reduce M1 marker expression and increase M2 marker expression in microglia. This finding supports the idea that IRF1 is capable of participating in anti-inflammatory processes in a highly specific manner, and its role may be plastic and dependent on both cell type and context (81). Furthermore, in a model of calcium oxalate nephrocalcinosis, it was shown that activation of the aryl hydrocarbon receptor (AhR) was able to slow disease progression through a shift from M1- to M2-type macrophage polarization. For example, AhR was shown to transcriptionally increase miR-142a, which targeted and inhibited the expression of IRF1 and HIF-1α, thus facilitating a shift toward M2-like macrophages (46). Thus, this pathway provided compelling evidence that targeted downregulation of IRF1 is permissive for the induction of IL-4/IL-13 responsive M2 marker genes.

Together, these studies further establish IRF1 as the master negative regulator of M2 polarization. IRF1 is integrated into regulatory networks that inhibit the transcription of M2 marker genes through its antagonism of the STAT6 signaling pathway. Inhibitory miRNAs often mediate the relief from this inhibition and will be necessary for the full activation of the M2 polarization program.

### 4.2 Key signaling nodes regulating M2 polarization

IRF1 has significant effects on suppressing M2 macrophage activation due to its ability to disrupt canonical pathways that control alternative activation of M2 macrophages. One way it accomplishes this is by inducing feedback signaling molecules (including SOCS1) through an IRF1 dependent mechanism that inhibits the JAK-STAT6 signaling pathway, which is the classical pathway by which IL-4/IL-13 cause the polarization of M2 macrophages. Therefore, the blockade of JAK-STAT6 signaling through SOCS1 driven by IRF1 provides an important, upstream mechanism to prevent pro-M2 signals from being expressed (64). This regulatory interaction occurs across many disease states; calcium oxalate nephrocalcinosis is one example, where AhR signaling suppresses M1 polarization and promotes M2 polarization by downregulating IRF1 to thereby serve as an important “off switch” for the M2 macrophage phenotype (46). The research indicates that methionine restriction decreases renal ischemia-reperfusion injury via global inhibition of macrophage polarization, which correlates with decreased epigenetic modifications at the Irf1 promoter as well as subsequent down regulation of IRF1 expression level which, in turn, reinforces the influence of IRF1’s transcription regulation on polarization fate (41). This intricate regulation of IRF1 establishes it as a transcription hub that defines the balance between inflammatory and reparative macrophages through either splicing or modulating the actions of various M2 differentiation pathways.

In oncological research, loss or reduction of IRF1 appears to be strongly linked with increased levels of M2-like TAMs, which suggests that IRF1 is an important barrier to macrophages being transformed into a pro-tumorigenic phenotype within the tumor microenvironment (TME). There are many established associations between IRF1 and M2-like TAMs in glioblastoma (GBM). For example, when PTEN-deficient glioma cells activate the AKT-GSK3β-IRF1 pathway, they secrete large amounts of galectin-9 (Gal9), which then terminally M2 polarizes macrophages through Tim-3 receptors (82).

Similarly, IRF1 is stimulated by tumor-derived factors to promote M2 polarization in macrophages. For example, high levels of miR-106b-5p have been found in glioblastoma-infiltrating macrophages, leading to decreased levels of IRF1 and thereby promoting M2 polarization via inactivation of the IRF1/IFN-β signaling pathway (42). In lung cancer, hypoxic tumor cells produce exosomes containing miR-21 that down-regulate IRF1 expression in macrophages, leading to M2 polarization and increased tumor growth and progression (44). The same principle applies in colorectal cancer (CRC), where radio-resistant CRC cells overexpress miR-1226-5p to promote tumor aggressiveness and M2 macrophage polarization through down-regulation of IRF1 (76). SUMOylated nuclear IL-33 in human hepatocellular carcinoma (HCC) cells is associated with increased stability of IRF1 and, surprisingly, increased PD-L1, thus promoting M2 macrophage polarization and immune evasion (78). In addition, in advanced HCC, IRF1 stimulates DNA damage response in tumor cells, resulting in increased MICA expression via IRF1. MICA is a ligand for NKG2D and a receptor on NK cells that activates them. MICA+ cells produce PROS1, which binds with the AXL receptor and activates macrophage MMP9, which can lead to the M2-like phenotype of macrophages and subsequent immune escape (83). Collectively, this body of evidence from multiple types of malignant disease firmly supports the notion that IRF1 is a major barrier to the M2 polarization of TAMs. The dysfunction of IRF1, which can occur by many different tumor-derived means, is a key event that leads to an immunosuppressive and pro-tumorigenic tumor microenvironment.

## 5 Interaction between immunometabolic reprogramming and IRF1 in macrophages

### 5.1 Regulation of metabolic pathways associated with M1

IRF1 is essential for activating macrophages toward an M1 (pro-inflammatory) phenotype mainly through the reprogramming of glucose metabolism to allow aerobic glycolysis (the Warburg effect) and the pentose phosphate pathway. This metabolic rewiring is necessary in order for activated macrophages to meet the biosynthetic and functional needs of these cells. Induction of IRF1 is also necessary for the expression of glycolytic enzymes needed to establish the metabolic profile of M1 macrophages because IRF1 is under the control of the downstream effects of the IFN-y/JAK-STAT1 pathway (67). An example of this is the use of flavonoids (such as fisetin) to inhibit IRF1 activity in IFN-y stimulated macrophages, which reverses the glycolytic switch that occurs in M1 macrophages and downregulates pro-inflammatory genes that are induced by IFN-y, restores mitochondrial oxidative phosphorylation (OXPHOS) and decreases lactate production, all effects that are aligned with the inhibition of IRF1 (67).

In addition, nutrition impacts the degree of control exercised over the polarization of macrophages (M)/; dietary methionine restriction inhibits M1 and M2 macrophage polarization processes through suppression of IRF1 expression (37). Methionine availability is epigenetically linked to IRF1 dependent transcription and signifies IRF1 as a metabolic sensor that translates nutritional information into the regulation of macrophage inflammatory pathways.

Apart from its impact on glycolysis, IRF1 also plays an important role in promoting the M1 phenotype by enhancing mitochondrial functional outputs, including the generation of reactive oxygen species (ROS). The role of ROS in M1 macrophages is twofold; 1) the generation of ROS is essential for destroying pathogens and thus; 2) ROS also serve as second messengers to promote transcriptional events associated with inflammation. An IRF1-dependent decrease in oxidative phosphorylation (OXPHOS) as measured by decreased oxygen consumption rate (OCR) in cells exposed to IFN-γ promotes the accumulation of ROS (67). IRF1 is also an important transcriptional regulator of inflammatory cell death as a consequence of mitochondrial dysfunction. In autoimmune disease, signals from Th1 cells induce pyroptosis of M1 macrophages through an IRF1-dependent mechanism. In this instance, IRF1 binds to the cyp1 promoter to induce transcription of the casp-1 gene to initiate the highly inflammatory pyroptotic response (84). The data suggest IRF1 is not only a transcriptional regulator of metabolic enzymes but also a transcriptional executor of inflammatory responses in which ROS play an important role as signaling molecules.

IRF1, which has been found to play a significant role in regulating the immune system and metabolic processes, is becoming more evident through emerging research. Multi-omics studies have shown that IRF1 is a predictive immune biomarker in complex metabolic diseases (example: type 2 diabetes with tuberculosis), with the JAK-STAT and NF-κB networks being the main pathways affecting IRF1 function and leading to an active innate immune system composed primarily of M1 macrophages (85). As an example, intra-cytokine response (ICR) mediates the synergistic anti-inflammatory effects when given together docosahexaenoic acid (DHA) and dexamethasone in systemic lupus erythematosus (SLE) by inhibiting transcription factors (primarily IRF1) and promoting a phenotypic shift of M1 to resolvable M2 macrophages (86, 87). These results indicate that therapies targeting IRF1 and its downstream pathways represent a novel approach to alter macrophage immunometabolism, thus providing evidence of a possible target for the treatment of inflammation and metabolic diseases.

### 5.2 Modulating M2-type metabolic adaptation

IRF1 inhibits M2 polarization by inhibiting the specific metabolic pathway used by M2 cells. M2 cells primarily generate energy through oxidative phosphorylation (OXPHOS) or fatty acid oxidation (FAO) unlike M1 cells. By inhibiting the activity of key FAO enzymes such as CPT1A and/or through the modulation of metabolic stress kinase such as AMPK, IRF1 alters the metabolic profile associated with M2 polarization.

Recent studies provide strong evidence of the interplay between metabolic state and macrophage function. For example, in a renal ischemia-reperfusion model of methionine restriction, this technique reduced the polarization ability of bone marrow-derived macrophages to both M1 and M2 phenotypes (37). This limitation also reduced the expression of Irf1 due to reduced H3K4me3 modifications at the Irf1 promoter, resulting in the inability to polarize these macrophages (37). This demonstrates that IRF1 acts as a key immunometabolic switch that is responsive to nutrition. Additionally, metabolic environments at the site of tissues dictate the activation state of macrophages. For instance, perirenal adipose tissue has a non-inflammatory milieu with increased expression of thermogenic genes, functional TCA cycles, and high levels of OXPHOS activity (88). On the other hand, renal sinus adipose tissue hosts a pro-inflammatory and macrophage-rich environment with very high expression of pro-inflammatory genes, including IRF1 (88). This provides evidence for how local metabolic environments regulate the macrophages’ activation state through factors like IRF1.

In a number of conditions, including systemic lupus erythematosus (SLE), the use of two or more drugs may lead to a change in the phenotype of an immune cell through modifying the metabolic and inflammatory pathways. The combination of DHA and dexamethasone (DEX) has been shown to attenuate lipopolysaccharide (LPS)-induced inflammatory gene expression in macrophages derived from SLE-prone mice (86). The use of these two compounds has been shown to lead to the activation of pro-resolving metabolic pathways and to inhibit LPS-induced inflammatory pathways and thereby resulted in a marked shift toward an M2 macrophage phenotype (86). The therapeutic effect of DHA and DEX appears to be caused, in part, by their inhibition of the transcription factor IRF1, which is involved in the regulation of inflammatory responses (86, 87). Targeting IRF1 and the associated metabolic pathways may provide a therapeutic opportunity for producing an anti-inflammatory states.

When a patient has both T2DM and TB, they show signs of immune/metabolic dysfunction because IRF1 is a central biomarker linking these two systems (85). Relatedly, functional studies in such patients find that imbalance (due to overactivation of innate immune cells [M1/M2 ratio] and under activation of adaptive immune cells) of the immune system is the result of several dysregulated immune pathways (Toll-like receptor, NF-κB, and JAK-STAT), which therefore would reinforce the finding that IRF1 inhibits M2 macrophage polarization through inhibiting key metabolic reprogramming processes (OXPHOS and FAO), thereby establishing an association between transcriptional regulation, cellular metabolism, and immune function. As shown in **Figure 3**, IRF1 serves as a transcriptional regulator and acts as a pioneer factor by altering chromatin accessibility to link the immunometabolism of macrophages with their epigenetic programming to determine macrophage polarization fate.

**Figure 3:**
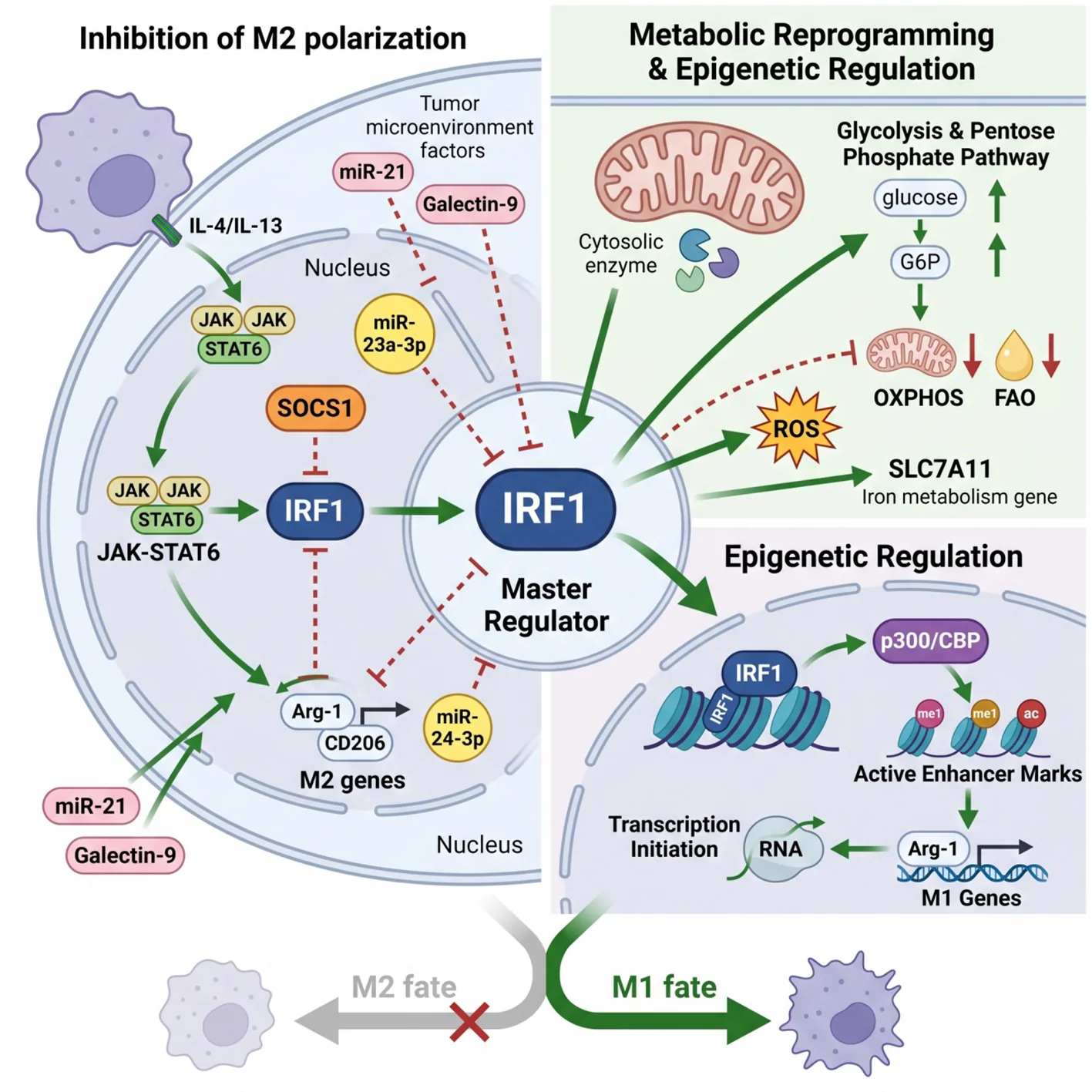
IRF1 antagonizes M2 polarization and integrates immunometabolic reprogramming with epigenetic regulation.

This schematic illustrates how IRF1 antagonizes alternative (M2) macrophage polarization and coordinates immunometabolism. Left panel (Inhibition of M2 polarization): (1) IRF1 transcriptionally activates SOCS1, which subsequently inhibits the JAK-STAT6 cascade, thereby blocking IL-4/IL-13-induced M2 polarization. (2) Specific miRNAs (e.g., miR-23a-3p, miR-24-3p, and miR-106b-5p) target Irf1 mRNA, relieving its suppression and enabling the expression of M2 marker genes (Arg-1, CD206, TGF-β, and IL-10). (3) Within the tumor microenvironment, tumor-derived factors (such as miR-21, miR-1226-5p, and galectin-9) suppress IRF1 to facilitate M2 polarization. Right panel (Metabolic reprogramming): IRF1 drives aerobic glycolysis (the Warburg effect) and the pentose phosphate pathway, while simultaneously suppressing oxidative phosphorylation (OXPHOS) and fatty acid oxidation (FAO). Additionally, IRF1 modulates mitochondrial ROS production and regulates ferroptosis-related genes (SLC7A11, GPX4). Bottom panel (Epigenetic regulation): Acting as a pioneer factor, IRF1 binds to compacted chromatin and recruits p300/CBP alongside chromatin remodeling complexes. This recruitment establishes active histone marks (H3K4me1, H3K27ac) and facilitates *de novo* enhancer formation. Arrows indicate activation or promotion; T-bars indicate inhibition.

## 6 The epigenetic regulatory role of IRF1 in macrophage polarization

### 6.1 As a “pioneer factor” for chromatin modification

IRF1 (Interferon Regulatory Factor 1) is recognized as a pioneer transcription factor, which plays a fundamental role in initiating the inflammatory gene program in macrophages in response to a stimulus. Because of its pioneering ability to bind to condensed or inactive chromatin domains, IRF1 induces a series of epigenetic changes that lead to the transcriptional activation of these genes. A recent study showed that TLR4 activation of IRF1 in human macrophages is necessary to promote opening of ISG (interferon-stimulated gene) loci, which is an important first step in the ISG response (89).

Histone acetyltransferases (e.g., p300/CBP) and chromatin remodeling complexes are recruited to the appropriate gene via IRF1 in the initial opening of chromatin at low-accessibility regions in macrophages in response to LPS and/or IFN-γ stimulation (33). At these loci, IRF1 works in coordination with either ISGF3 (induced by type I IFN) or GAF (induced by type II IFN) to create *de novo* enhancers that can be identified by active histone marks such as H3K4me1 (33). The ability of IRF1 to work cooperatively with other transcription factors, including STAT1 and NF-κB, is critical to its pioneer function. ChIP-seq results show that IRF1 binds to the promoters of target genes, including its own gene (Irf1) and to the promoter of STAT1, establishing a positive feedback mechanism of amplification of M1 polarization signals (52).

Multi-omics studies confirm that IRF1 plays a key role in determining the chromatin landscape. In ATAC-seq analysis of macrophage polarization, IRF1 was the most abundantly represented transcription factor found within M1 macrophages, and the IRF1 binding motifs were enriched in the newly created accessible chromatin at this time (64). Importantly, this epigenetic reprogramming does not appear to be a temporary phenomenon. In mycobacterial infection models, systemic inflammation leads to invariant epigenetic modifications—specifically, increased accessibility of chromatin at the Irf1 locus—which lasts from the level of the hematopoietic stem cell to that of the differentiated monocyte, thus creating central trained immunity (90).

The pioneer activity of IRF1 is closely linked to upstream signals and cellular metabolism. For example, methionine restriction in macrophages reduces the abundance of the active histone mark H3K4me3 at the Irf1 promoter, leading to decreased IRF1 expression and impaired polarization. This demonstrates a direct link between metabolite availability and IRF1-mediated chromatin regulation. Alternatively, the highly conserved long non-coding RNA CARINH regulates the expression of its antisense gene IRF1 in macrophages; its deletion decreases IRF1 expression, thereby disrupting target gene networks and increasing susceptibility to viral infection (37). In conclusion, the pioneer activity of IRF1 is fundamental to a competent immune response (48).

A total lack of IRF1 in humans causes life-threatening susceptibility to mycobacterial infections due to a major defect in IFN-γ-activated macrophage immunity; this emphasizes the importance of IRF1 in the process of unlocking and activating important clusters of host defense genes (31).

### 6.2 Interaction with non-coding RNA networks

The expression and activity of IRF1 are finely tuned by a complex system of non-coding RNAs (ncRNAs) such as microRNAs (miRNAs) and long non-coding RNAs (lncRNAs), with GI being regulated by IRF1 itself, setting up complicated feedback and feed-forward loops that define macrophage fate. A key regulatory mechanism is the direct or indirect post-transcriptional repression of IRF1 by miRNAs.

For example, miR-130b-3p downregulation in M1-polarizing conditions inhibits M1 macrophage polarization by directly targeting the 3’-UTR of Irf1 mRNA to repress IRF1 expression, thereby inhibiting the expression of downstream targets that drive M1 macrophage polarization such as CCL5, CXCL10, iNOS, and TNF-α (40). Furthermore, miR-23 was delivered via exosomes from adipose-derived stem cells (ADSCs) to inhibit M1 polarization by directly suppressing IRF1 expression (91). In the context of cancer and therapeutic resistance, miR-1226-5p is upregulated in radioresistant colorectal cancer (CRC) cells and delivered to macrophages to inhibit IRF1, thus promoting M2 polarization and increasing tumor aggressiveness (76). Additionally, miR-21 from hypoxic tumor-derived exosomes drives M2 polarization and promotes lung cancer progression by targeting IRF1 (44). Finally, other miRNAs such as miR-106b-5p in glioblastoma-associated macrophages promote M2 polarization by inhibiting IRF1 and its downstream IFN-β signaling cascade (42).

On the other hand, the expression of IRF1 is also reduced in an indirect manner when upstream activators of IRF1 are targeted by miRNAs. One example is miR-103, which inhibits the M1 polarization of macrophages by targeting and blocking the activity of STAT1, and this results in decreased activity of the entire STAT1/IRF1 signaling pathway (43). In addition, regulatory networks targeting IRF1 also exist that involve transcription factor-mediated expression of miRNAs. That is, Nrf2 upregulates the expression of miR-93-5p, which then targets both TLR4 and Irf1 mRNA. This regulation ultimately forms an Nrf2/miR-93/TLR4/IRF1 Axis that inhibits the M1 polarization of macrophages, therefore reducing inflammatory damage (45). Furthermore, the transcriptional activation of miR-142a by AhR results in the downregulation of both IRF1 and HIF-1α by the targeting of their respective 3’-UTRs. This ultimately blocks M1 macrophage polarization and enhances M2 macrophage polarization (46).

The regulatory network also includes a number of lncRNAs that will actively participate as well as miRNAs. For example, the lncRNA MRF (MCP1 regulatory factor) is transcriptionally induced within mesenchymal stem cells (MSCs) by IRF1, following early co-culture with monocytes. Following this, MRF acts as a pro-inflammatory agent by binding to heterogeneous nuclear ribonucleoprotein D (HNRNPD) to upregulate monocyte chemotactic protein-1 (MCP1) and thereby regulate the recruitment of monocytes and the polarization of macrophages (92). This serves as an example of a paradigm in which IRF1 transactivates a lncRNA which acts as a mediator of immune function. In addition, IRF1 can bind and regulate the expression of circulating miRNAs associated with disease, such as those involved in myasthenia gravis, and as such, can be considered to have a significant overarching role in the regulation of ncRNAs (93).

These regulatory networks are also regulated, directly or indirectly, by circular RNAs (circRNAs). circSLC43A1 antagonizes the inhibitory effect of miR-1226-5p on IRF1, thus diminishing M2-polarizing and pro-tumorigenic activity from this miRNA to create radioresistant CRC (76). This adds an additional mechanistic level of complexity to the overall picture. Moreover, super-enhancer-mediated transcription (e.g., BRD4/p300) can induce M2-polarizing miRNAs such as miR-146a-5p; however, it should be noted that the interaction between miR-146a-5p and IRF1 was not specifically evaluated in the referenced study. Nevertheless, these data demonstrate the complex interplay between epigenetic and transcriptional mechanisms regulating miRNA expression, which is likely to involve IRF1 signaling pathways (80).

In terms of therapeutics, small extracellular vesicles (sEVs) produced by TNF-α-activated menstrual stem cells contain higher levels of miR-24-3p than sEVs from non-stimulated stem cells. Importantly, miR-24-3p promotes M2 polarization through the downregulation of colonic IRF1, enhancing sEVs’ functionality in colitis (58). Bone marrow-derived exosomes (BMSC-Exos) also promote M2 polarization in tendon-bone junction healing via the delivery of miR-23a-3p that targets IRF1 (57). Furthermore, bioinformatics analyses have further supported the master regulatory role of IRF1 in directing the transcription of chemokines that influence macrophage polarization in a variety of disease processes, including atherosclerosis (94).

The interactions between IRF1 and ncRNAs would create a highly intricate network that can be classified as bidirectional. Consequently, the ultimate final outcome of the ILF1-regulated macrophage phenotypes will be determined by the pro-inflammatory cytokines produced by these same macrophages via the interaction of their ncRNA components.

## 7 Pathophysiological significance of IRF1 in regulating macrophage polarization

### 7.1 Role in anti-infective immunity

IRF1plays a key role in protecting the host from a variety of pathogens (especially intracellular pathogens), mainly through mediating the activation of M1-type macrophages. The importance of IRF1 is shown by genetic studies conducted in humans; individuals who are deficient in IRF1 due to inherited mutations have increased susceptibility to develop severe infections caused by mesophilic mycobacteria and other bacteria that normally reside within macrophages. These data provide evidence of the unique contribution that IRF1 plays in mediating IFN-γ-driven immunity within these cells (31). On the other hand, when the IRF1 gene was genetically deleted in a mouse model of sepsis-induced ARDS caused by cecal ligation and puncture, deletion of the IRF1 gene was shown to greatly reduce inflammatory lung injury while at the same time reducing loss of M1-polarized macrophage infiltration into the lung, although this reduction was associated with a reduction in bacterial clearance (52).

As indicated by research on human metapneumovirus (HMPV), Chikungunya Virus (CHIKV), and African Swine Fever Virus (ASFV), IRF1 has a major relevance to antiviral immunity. The key to the antiviral response following HMPV infection of human macrophages is the induction of IRF1, which occurs via TBK1-mediated and type I interferon-mediated signaling (23). In CHIKV-infected macrophages, TLR3/TRIF-mediated IRF1 activation is necessary to induce the IL-27p28 subunit, resulting in the establishment of a robust, IFN-independent antiviral and inflammatory response (95). In addition, the antiviral agent Toosendanin inhibits ASFV replication by stimulating host IFN-stimulated gene Irf1 expression in porcine alveolar macrophages. Further emphasizing its importance is IRF1’s evolutionary conservation; teleost fish express IRF1 homologs in response to the viral mimic poly(I:C), and these proteins are responsible for the orchestration of antiviral responses via their induction of various subclasses of type I interferons (96).

The role of IRF1 in effective host defenses is two-fold: IRF1 helps produce effective antimicrobial effector molecules and connects the innate and adaptive immune systems. A major point in this continuum is the ability of IRF1 to mediate the inducible nitric oxide synthase (iNOS) transcriptional program leading to high levels of NO production, a potent mediator of intracellular pathogen clearance through a series of feed-forward regulatory loops. In a septic ARDS model, for example, IRF1 binds to the promoter region of STAT1 and initiates STAT1 transcription, thereby creating a positive feedback regulatory loop between IFIH1, IRF1, and STAT1 that dramatically amplifies M1-polarizing signals (52). In a similar manner, by promoting phosphorylation of STAT1 in response to IFN-γ, the transcription of Irf1 is increased, enhancing downstream ISG expression and production of pro-inflammatory cytokines and chemokines (70).

IRF1 activity is regulated by non-coding RNAs that modulate how IRF1 responds to bacterial or viral charges. The lncRNA (long non-coding RNA) CARINH (or its homologous forms) is induced in macrophages that are infected with viruses, being required for keeping IRF1 levels high; knock-down of CARINH decreases the IRF1 target gene network and makes the cells susceptible to viral infection (48). Conversely, many organisms (e.g., viruses and bacteria), have evolved mechanisms to manipulate the immune system during infection. For example, Listeria monocytogenes affects host autophagy through own proteins (e.g., RNF144A) that mark (ubiquitinate) proteins of the autophagic machinery (e.g., BECN1); thus, the ongoing evolutionary arms race between the pathogen’s mechanisms for surviving and the host’s methods for protecting itself is visually evident (97).

In summary, IRF1 is a key integrator, coordinating inputs from PRRs (such as TLRs) and cytokine receptors (such as IFNs) to activate the complete program of genes critical for fighting infections. By activating, among others, iNOS, the pro-inflammatory cytokines TNF-α and IL-6, and the chemokines CCL2 and CXCL10, IRF1 plays a role in two ways; IRF1 functions directly to eliminate the pathogen, and activates/recruits various subsets of immune cells, providing a vital link between innate and adaptive immunity (98, 99).

### 7.2 Dual role in tumor immunity

In a tumor’s microenvironment, tumor-associated macrophages usually show an M2 phenotype. In addition to causing blood vessels to grow and tissues to remodel, they also generate significant immunosuppression. As a result, IRF1, an important regulator of pro-inflammatory and anti-tumor effects, is often down-regulated or functionally down-regulated in a tumor microenvironment. However, the impact of IRF1 in tumors is highly dependent on context. In HCC, the SUMOylated form of interleukin-33 (IL-33) increases the nuclear stability of IRF1. Ironically, stable IRF1 increases the amount of PD-L1, causes M2 polarization of macrophages and enhances immune evasion (78). The AKT-GSK3β-IRF1 pathway is activated by the loss of PTEN in glioblastoma (GBM), resulting in the secretion of galectin-9 (Gal-9), which activates the Tim-3 pathway and leads to M2 macrophage polarization. This contributes to tumor growth and angiogenesis (74). Additionally, the immunosuppressed nature of the hypoxic tumor microenvironment (TME) in lung cancer (LC) is further enhanced by tumor-through exosomal mRNA downregulating IRF1 in macrophages, causing them to be skewed to an M2 phenotype and promote the proliferation of cancer cells (44). This trend is also seen in other solid tumors. In ovarian clear cell carcinoma, a malignancy usually indicating a poor prognosis, the TME is significantly populated with tumor-associated macrophages (TAMs), but has a severe deficiency of T cells and lacks any significant proportion of IRF1-positive M1-TAMs (65). These findings suggest that the tumor microenvironment (TME) often suppresses IRF1 expression or utilizes downstream signaling pathways of IRF1 to create an environment that sustains tumorigenesis and enhances immunosuppression.

Alternatively, when IRF1 is restored or overexpressed in macrophages then they will be able to differ as much as possible from their pro-tumor M2 phenotype and are able to develop an effective anticancer phenotype similar to M1. This change will improve their phagocytic capabilities, increase their cancer cell-killing capabilities, and decrease the effects of TME immunosuppression. Since the TME is important for tumors to grow, it has been shown that artesunate (AS) can act on the TME and reverse the direction in which the TME acts to support the growth of non-small cell lung cancer (NSCLC) via a STAT1/IRF1 mediated repolarization of M2-like to M1-like via TME acquisition by macrophages, enhancing phagocytosis, and activation of T-cells when used in conjunction with anti-PD-1 therapies (69). AS can also be employed to achieve similar M1 reprogramming of a macrophages from M2 in triple-negative breast cancer (TNBC) through the use of celastrol-loaded M1 macrophage-derived exosomes wherein this strategy will downregulate MHC-I expression on the tumors using the IRF1-CIITA pathway resulting in the tumor cells becoming highly susceptible to macrophage phagocytosis (100).

Also, by using targeted interventions against negative feedback loops downstream from IRF1, we are able to significantly improve our ability to generate anti-tumor immunity. A CRISPR-Cas9 screen has shown that when IRF1 induces SOCS1, it limits the ability to maintain expressing CXCL9, which is a T-cell chemoattractant for T-cells. Therefore, by selectively inhibiting SOCS1, CXCL9 expression can be significantly potentiated for intratumoral macrophages leading to a significant increase in efficacy of immune checkpoint blockade (101). In skin cutaneous melanoma (SKCM), IRF1 is a central hub gene for a tumor immune microenvironment-associated signature (TIMAS) associated with strong T-cell infiltration, M1 macrophage enrichment, and immunotherapy response (102)99^[77]^. Collectively, these results strongly support that stimulating the IRF1 axis with various approaches represents an exciting new therapeutic strategy for re-programming TAMs to re-invigorate anti-tumor immune responses and to overcome therapeutic resistance.

IRF1’s involvement in tumor immunity is complex and not straightforward. For example, in HNSCCs, IRF1-mediated inflammatory responses (such as IFN-γ and IRF1) have been associated with increased tumorigenesis and malignancy, especially when prolonged/chronic inflammatory response has a role in development of HNSCCs, as described above. To illustrate this point further, Aim2−/− mice had increased levels of IFN-γ and IRF1 than either WT or Aim2+/− mice; increased M1:M2 ratio; higher levels of adaptive immune signatures (as assessed by multiplex immunohistochemistry); etc., suggesting that AIM2, as a cytosolic DNA sensor, is typically a negative regulator of both IFN-γ and IRF-1 production or activity and that increased hyperactivation of IRF1 in specific situations can ultimately lead to tumor promotion (103).

IRF1 is associated with a favorable response to radiotherapy in addition to CD8+ T cells and M1 macrophages, which are found within a ‘hot’ immune microenvironment (104), Sustained induction of IRF1, however, can create deleterious effects in late-stage hepatocellular carcinoma (HCC) tumors. In the case of HCC, therapy-induced DNA damage within the tumor cells triggers substantial upregulation of IRF1, leading to increased MICA and PROS1 expression. PROS1 that is released activates macrophage-derived MMP9 through the AXL receptor, which cleaves MICA from the surface of the tumor, creating soluble MICA (sMICA) that actively promotes immune evasion (58, 80, 83). This elegant example illustrates how advanced tumors exploit IRF1 signaling to ultimately paralyze the effector function of NK and T cells. In a different context, however, a study of cervical cancer demonstrated that de-expression of IRF1 and other ISGs was significantly associated with a high-risk gene signature for distant metastasis, corresponding with a ‘cold’, immune-excluded TME (105). The absence of IRF1-dependent inflammation is strongly correlated with metastatic progression, suggesting that both the complete absence of IRF1 and the chronic hyperactivation of the IRF1 response may be pathologically detrimental.

Beyond its well-documented function in driving M1-like responses, IRF1 also shapes disease progression by restraining pathological M2-like macrophage programming. Sustained downregulation of IRF1 removes this inhibitory constraint, permitting aberrant M2-like activation that is frequently observed in the tumor microenvironment and various fibrotic disorders. In metabolic kidney injury, specifically calcium-oxalate nephrocalcinosis, dysregulated signaling pathways involving IRF1 contribute to the polarization of macrophages toward an M2-like phenotype, which may influence crystal phagocytosis and stone development (106). Despite its canonical role in suppressing alternative activation, IRF1 can exert context-dependent, paradoxical pro-M2 effects under specific pathological conditions. For instance, emerging evidence suggests that specific post-translational modifications and upstream signals can stabilize IRF1 in ways that paradoxically promote M2-like polarization in certain malignancies (107).

Consequently, IRF1 does not have a completely negative effect on tumors; the outcome of the variable interactions between IRF1 and the TME will dictate the overall function, as spatiotemporal dynamics, cellular context, and crosstalk with other pathways all impact the role of IRF1 in regulating the TME. Therefore, finding a delicate balance among these interactions will be crucial for achieving effective immune-mediated tumor clearance through IRF1. The context-dependent beneficial and detrimental effects of IRF1 in multiple diseases are summarized in **Table 2**.

**Table 2:** Therapeutic strategies based on IRF1 modulation.

IRF1-agonist strategies (activate IRF1 for therapy)				
Infectious diseases				
**Disease/model**	**Functional role of IRF1**	**Downstream effects**	**Targeting strategy**	**References**
Mycobacterial infection	Protective: promotes M1, bactericidal activity	iNOSvi NOOS pathogen clearance	Agonist (IFN-γ)	(31, 108)
Viral infection (HMPV, CHIKV, ASFV)	Protective: induces ISGs and interferons	IRF1r → IFN-β/λ↑, viral replicationI	Agonist (TBK1 agonist)	(22, 95, 98)
Cancer				
**Disease/model**	**Functional role of IRF1**	**Downstream effects**	**Targeting strategy**	**References**
Non-small cell lung cancer (NSCLC)	Protective: reprograms TAMs to M1	STAT1/IRF1-mediated TAM repolarization, T-cell activationt	Agonist (artesunate)	(69)
Ovarian cancer	Protective: CXCL10^+^ IRF1^+^ M1 TAMs	Antitumor immunity, better therapy response	Agonist	(65)
Colorectal cancer	Protective: upregulates CXCL10, recruits CD8^+^ T cells	CXCL10ts CD8^+^ T-cell infiltrationTE tumor growthr	Agonist	(66)
Melanoma	Protective: SOX10-IRF4-IRF1 axis	PD-L1↑ (sensitizes to immunotherapy)	Agonist (HDAC inhibitor)	(109, 110)
IRF1-antagonist strategies (inhibit IRF1 to curb inflammation)				
Autoimmune/autoinflammatory diseases				
**Disease/model**	**Functional role of IRF1**	**Downstream effects**	**Targeting strategy**	**References**
Sepsis (CLP model)	Pathogenic: drives inflammatory lung injury	IFIH1/IRF1/STAT1 axis → M1is lung injuryI	Inhibitor (fludarabine)	(52, 68)
Sepsis-induced cardiomyopathy	Pathogenic: promotes pyroptosis and inflammation	IRF1 → Caspase-1↑ → pyroptosiso	Inhibitor (NAC, siRNA)	(111)
Osteoarthritis	Pathogenic: promotes M1 and chondrocyte pyroptosis	IRF1 → NLRP3t → pyroptosise	Inhibitor	(39, 112)
Atherosclerosis	Pathogenic: drives M1 polarization	SUB1 → IRF1 → pro-inflammatory genesa	Inhibitor (myeloid-specific Sub1 KO)	(38)
Autoimmune myocarditis	Pathogenic: Th1-induced M1 pyroptosis	IRF1 → Caspase-1↑ → pyroptosisd	Inhibitor (biomimetic nanoparticle-delivered siIRF1)	(84)
Psoriasis	Pathogenic: promotes inflammation	IRF1/GSTM3 axis	Inhibitor (celastrol)	(113)
Calcium oxalate nephrocalcinosis	Pathogenic: promotes M1 polarization	TLR4/IRF1 pathway	Inhibitor (sulforaphane, AhR agonist)	(45, 46)
Cancer (dual or pathogenic IRF1 – antagonist approaches)				
**Disease/model**	**Functional role of IRF1**	**Downstream effects**	**Targeting strategy**	**References**
Hepatocellular carcinoma (HCC)	Dual: IRF1 stabilization promotes immune escape	SUMOylated IL-33 stabilizes IRF1 → PD-L1↑, M2 polarization	Inhibitor (IL-33 SUMOylation inhibitor)	(78)
Glioblastoma (GBM)	Pathogenic: Gal-9 drives M2 polarization	PTEN loss → AKT-GSK3β-IRF1 → Gal-9↑ → M2↑	Inhibit Gal-9/Tim-3	(82)

CLP, cecal ligation and puncture; TAM, tumor-associated macrophage; KO, knockout; NAC, N-acetylcysteine; AhR, aryl hydrocarbon receptor.8 Using IRF1 to treat diseases: what we can do and what is hard.

Bold text denotes column headings and category labels.

### 8.1 Making IRF1 stronger to fight infection and tumors

Pharmacological activation of IRF1 represents a promising strategy to reprogram pro-tumorigenic macrophages into anti-tumor M1-like phenotypes. This is primarily because IRF1 orchestrates the expression of gene networks essential for host defense and inflammatory responses—key hallmarks of the M1 phenotype. For instance, in human primary macrophages infected with *Mycobacterium avium*, an autocrine/paracrine TNF-α and IL-6 signaling loop is required to trigger IRF1 nuclear translocation. This subsequently induces downstream targets such as *IRG1*, which produces the antimicrobial metabolite itaconate to restrict intracellular bacterial growth (108). These findings underscore the non-redundant role of IRF1 in IFN-γ-dependent macrophage immunity against mycobacteria and related intracellular pathogens. This is further corroborated by clinical evidence where complete IRF1 deficiency in humans leads to severe mycobacterial disease, while antiviral IFN-α/β immunity remains largely functional (31).

IRF1 Activation is able to undermine the various mechanisms in place by which tumors evade immune destruction. As an example, in HCC, DNA damage or inhibition, of CHK1 leads to an upregulation of IRF1 levels, transcriptionally activating the expression of MICA. This promotes the ability of NK cells and CD8+ T cells to infiltrate the tumor and enhance anti-tumor immunity (114). Similarly, in NSCLC, CGA (a natural product) inhibits the expression of PD-L1 induced by IFN-g via an inhibition of the p-STAT1/IRF1 axis, which enhances T cell activity and increases the effectiveness of anti-PD-1 therapies (115). In melanoma, targeting the SOX10-IRF4-IRF1 will upregulate the expression of IRF1 and PD-L1 independent of the canonical JAK-STAT pathway and may thus serve as a means to “warm up” those tumors that are considered immunologically cold, such that the responsiveness of those tumors to immune checkpoint blockade may be improved (109). In conclusion, these findings indicate that agents with the capacity to stabilize the IRF1 protein, promote nuclear translocation of the IRF1 protein, or facilitate transcription of the IRF1 gene will shift the balance of macrophage functionality toward a tumoricidal state and, thus render tumors more immunogenic and susceptible to therapeutic intervention.

IFN-gamma has potential as a method of enhancing IRF1 through a direct administration approach, but the caution one has with this approach is also in regards to the possibility of systemic inflammatory side effects due to the use of the IFN-gamma molecule. IFN-gamma induces IRF1 via the JAK-STAT signaling pathway in murine macrophages; in addition to being a potent inducer of IRF1, it also up-regulates the expression of Samhd1 through an IRF1 dependent mechanism by releasing a repressor complex from the promoter of Samhd1 (32). However, while IFN-gamma has the ability to promote antigen presentation within a tumor environment by inducing CD47 expression (to interact with the SIRPα receptor on macrophages to inhibit/impede phagocytosis), the IFN-gamma pathway is also a “double-edged sword”; therefore, IFN-gamma, when administered, can also, via CD47, mediate immune escape (116).

In order to avoid these risks from the immune system as a whole there is a need for a more targeted way to activate the beneficial arm of the immune system through stimulating IRF1. In the case of colorectal cancer, the loss of the tumor suppressor (APC) can lead to the cancer avoiding detection by the immune system through PTPN13 regulation of STAT1 during IRF1 down-regulation through dephosphorylation. The use of peptides that mirror the C-terminus of APC (APC11) disrupts the PTPN13-STAT1 interaction and allows for proper signaling through STAT1 and IRF1, increasing the therapeutic response with a greater degree of specificity than using a systemic administration of IFN-γ alone (117). In ovarian cancer, antidepressant use was demonstrated to reverse the effects of chronic stress-associated depression on intrinsic IRF1 and the associated decrease in anti-tumor immune activity, restoring IRF1 expression and allowing immune cell infiltration into tumors (118). Collectively, the current understanding of the immunostimulatory properties of precision-based strategies, including the use of STAT1 activators, IRF1 stabilizers and the inhibition of negative regulators of IRF1 such as the E3 Ubiquitin Ligase SPOP (60, 119), indicates that these types of therapies will produce significant immunostimulatory effects while having a better safety profile compared to systemic cytokine therapy. The ultimate goal will be the identification and development of agents that specifically enhance the M1 transcriptional signature of IRF1 without causing widespread activation of systemic cytokine storms.

### 8.2 Inhibitor strategies: inhibiting IRF1 to alleviate excessive inflammation

Novel strategies for treating diseases associated with prolonged (or excessive) activity of M1 macrophages (e.g., autoimmune disorders like rheumatoid arthritis and inflammatory bowel disease, and sepsis) include the inhibition of Interferon Regulatory Factor 1 (IRF1). The rationale for targeting IRF1 is its identification as the main regulator of pro-inflammatory gene expression; this pathway can be inhibited to attenuate pathological inflammation and prevent damage to tissues. This prediction has been confirmed in a model of sepsis induced by cecal ligation and puncture (CLP) where IRF1 knockout mice demonstrate reduced lung injury and infiltration of M1 macrophages compared to wild-type controls (52). Likewise, administration of the STAT1 inhibitor fludarabine reduces levels of IRF1 protein and thereby blocks the alveolar infiltration by pro-inflammatory cytokines such as TNF-α, IL-6 and IFN-γ in LPS-induced acute lung injury (ALI) models through activation of the STAT1/IRF1 axis (68). Furthermore, in a model of sepsis-induced hepatic injury, coptisine demonstrates hepatoprotective effects through its binding to the STAT1 protein and inhibition of IRF1 activation; these actions have been shown to restore the expression of GPX4 and suppress ferroptosis of Kupffer cells (120). Promising results have also been reported for the use of IRF1 inhibition in other fields related to sterile inflammation and autoimmunity. For instance, celastrol exerts beneficial effects on psoriasis by targeting the IRF1/GSTM3 axis (113). Various strategies aimed at dampening TLR4/IRF1 pathway activation promote decreased M1 macrophage polarization and reduced inflammatory damage in models of calcium oxalate nephrocalcinosis (e.g., through the activation of the AhR-miR-142a axis or the administration of sulforaphane) (45, 46). Additionally, in the central nervous system, the knockdown of IRF1 expression in retinal microglia reduces LPS-induced inflammatory reactions by resulting in a shift from M1 to M2 marker expression profiles (77). BET inhibitors like JQ1 exert similar anti-inflammatory effects by reducing IRF1-mediated gene expression in human microglia (121).

An important problem with the potential therapeutic use of IRF1 inhibitors in clinical settings is how to target these agents to the appropriate cell type or tissue site. Delivery has to consider that systemic IRF1 inhibition may interfere with the normal functions of anti-infectious immune defenses and potentially impact hematopoietic stem cell maintenance and function (122). As a promising strategy, researchers are evaluating biomimetic nano-delivery systems. One example is the use of ZIF-8 nanoparticles to carry siIRF1 designed to downregulate IRF1 in a specific manner in M1 macrophages associated with autoimmune myocarditis; these nanoparticles are coated with a hybrid membrane combining T lymphocytes with macrophages. Researchers found that the delivery of siIRF1 using these nanoparticles downregulated IRF1 expression in M1 macrophages and prevented macrophage pyroptosis, thereby ameliorating the disease with limited systemic adverse effects (123). An alternative approach to target IRF1 relies upon utilizing endogenous microRNAs (miRNAs) as a means of regulating gene expression post-transcriptionally. Researchers have demonstrated that overexpressing miR-130b-3p, an miRNA that directly targets Irf1, inhibits colonic M1 macrophage differentiation and decreases lung inflammation in mouse models (40). In addition, exosomes derived from adipose-derived stem cells (ADSCs) and enriched with miR-23 have also been shown to inhibit M1 macrophage polarization through downregulation of IRF1, and thereby provide a protective mechanism against calcium oxalate stone formation (91).

In addition to providing temporal and contextual control, targeting the post-translational modifications of the IRF1 protein allows researchers to manipulate how and when this protein will be modified. The radiation-induced activation of IRF1 is one example; the NLS of IRF1 contains specific acetylation and phosphorylation sites, and the alteration of these sites inhibits the IRF1-mediated inflammatory death of cells (28). In addition, in atherosclerosis, myeloid-specific deletion of the IRF1 transcription coactivator SUB1 limits the induction of Irf1 gene expression and thus alters the macrophage phenotype from a pro-inflammatory M1-like state to a pro-resolving M2-like state (34). The key to success will be the development of delivery systems/inhibitors that can be “switched on” specifically within areas of inflamed tissue. Nevertheless, an important safety caveat must be considered for global IRF1-inhibitory therapies: broad suppression of IRF1 signaling may inadvertently de-repress M2-like transcriptional programs, which could exacerbate tissue fibrosis and other reparative-pathological side effects.

As shown in **Figure 4**, IRF1 exerts dual functions: its activation promotes antiviral and anticancer immunity, while its targeted inhibition alleviates tissue injury in autoimmune and hyper- inflammatory diseases.

**Figure 4:**
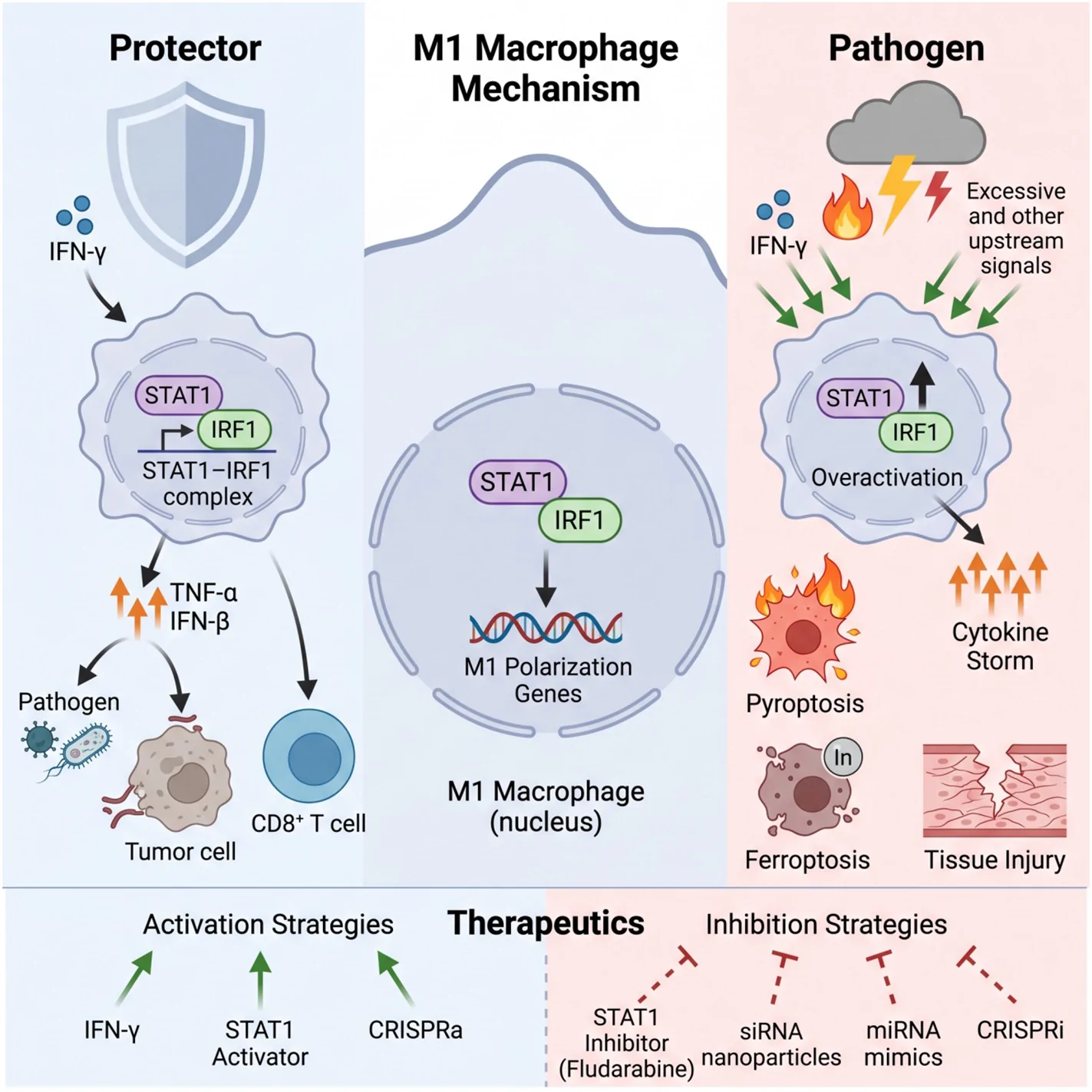
Dual roles of IRF1 in disease pathogenesis and therapeutic modulation.

This diagram describes both the pro- and anti-inflammatory properties of IRF1 as well as potential treatment approaches. In the left panel (Protector): In response to infections, IRF1 drives macrophages toward an M1 phenotype and produces pro-inflammatory cytokines like TNF-α and IFN-β, assisting in eliminating intracellular pathogens (e.g., mycobacteria and viruses). Within the cancer microenvironment, IRF1 assists with M1-like polarization of tumor-associated macrophages, production of CXCL10, and stimulation of CD8+ T-cells, which will improve the efficacy of immune checkpoint inhibitors. In the right panel (Pathogen): Autoimmune diseases or hyper-inflammatory diseases (e.g., septic shock, acute arthritis, acute atherosclerosis, myocarditis) are characterized by over-activation of IRF1, leading to uncontrolled M1 macrophages, cytokine storms, pyroptosis, and ferroptosis, all of which result in exacerbated tissue injury. In the bottom panel (Therapeutics): (1) Activators (green): Administration of IFN-γ, JAK/STAT1 activators, IRF1 -stabilizing PROTACs, and CRISPRa will be used to treat infections and tumors. (2) Inhibitors (red): STAT1 inhibitors (e.g., fludarabine), nanoparticle delivery of siRNA, miRNA mimics (miR-130b-3p, miR-23a-3p), CRISPRi, and PROTACs that degrade IRF1 are being explored for the treatment of hyper-inflammatory diseases. For the three approaches, arrows indicate activation and arrows with T-bars indicate inhibition.

## 9 Future research directions and technological prospects

### 9.1 Utilizing multi-omics technologies to decipher the IRF1 regulatory network

Integrating multi-omics technologies such as ChIP-seq and ATAC-seq and mapping the genome-wide binding landscapes of IRF1 in macrophages across multiple tissues and diseases has transformed our ability to target genes and networks related to IRF1. The application of single-cell RNA sequencing (scRNA-seq) has enabled the identification of unique subpopulations of macrophages based on their IRF1 expression patterns and functionally specialized states, as has been reported in human tonsils (124). In studies involving herpes simplex virus type 1 (HSV-1) brain infections, it has been demonstrated through the use of single-nucleus RNA sequencing that interferon-responsive microglia and macrophages possess an expanded IRF1 regulon relative to other cellular states (125). Additionally, spatial transcriptomic data are instrumental in determining the locations of individual IRF1 responsive genes as well as the locations of viral antigens within particular regions of the brain, providing essential spatial definition to the IRF1-mediated immune response (125). Likewise, scRNA-seq data have elucidated the role of IRF1 in the regulation of macrophage and microglial inflammation in tuberculous meningitis (126). Also, studies have examined crosstalk between tumor cells expressing MICA and macrophages expressing MMP9 in hepatocellular carcinoma and macrophage-promoted progression of osteoarthritis (83). Merging metabolomic and epigenomic information will ultimately provide an integrated view of how IRF1 integrates metabolic signals with epigenetic modifications to determine the fate of a macrophage. By employing epigenomic analysis (e.g., ATAC-seq), we demonstrate that the function of IRF1 is fundamentally connected to chromatin accessibility. How accessible chromatin is in IFN-gamma primed human macrophages lets us know how JAK inhibitors (JAKi) will affect the gene expression of a cell. Gene expression regulated by IRF1-STAT1 at locations where chromatin is accessible are inhibited by JAKi, while gene expression regulated by AP-1 and other factors are unaffected by JAKi (127). Further, IRF1 contributes to the development of innate immune memory by creating new accessible DNA regions and aiding in the production of new enhancers with ISGF3.

The relationship between IRF1 and cellular metabolism has been further clarified through metabolomic analyses. IFN-γ-induced IRF1-dependent alterations in nicotinamide metabolism lead to increased NAD+ production in RAW 264.7 macrophages and subsequently elicit a nitric oxide-dependent metabolic response (127). GAMG, a plant-derived natural product, has been shown to mitigate liver fibrosis by influencing the IRF1-SLC7A11 axis and thereby connecting the transcriptional effects of IRF1 to the ferroptotic death of inflammatory macrophages (33). In the case of ovarian cancer, chronic stress-related depression resulted in the silencing of IRF1 and as a result, dysregulation of lipid and glutathione metabolism, leading to immune suppression. The epigenetic reprogramming of airway macrophages in muco-obstructive lung disease modulates IRF1 activity and contributes to the inhibition of phagocytosis and sustained inflammation (128, 129). There also remain critical unresolved questions concerning how IRF1 modulates M2-like macrophage programming. First, additional myeloid-specific IRF1-knockout *in-vivo* animal models are urgently needed to dissect the cell-autonomous functions of IRF1 in restraining or permitting M2-like polarization, since much current evidence originates from correlative *in-vitro* studies. Second, high-resolution single-cell multi-omics approaches should be applied to profile IRF1-low M2-like macrophage subpopulations across diseased tissues and define their unique transcriptomic and epigenetic signatures. Altogether, these integrated multi-omics approaches suggest that IRF1 is a central molecular nexus that integrates cytokine signal input to facilitate the remodeling of epigenetic structure and functional networks to regulate macrophage effector functions during disease.

### 9.2 Development of specific tools for targeting IRF1

The creation of custom instruments that can accurately alter IRF1 is crucial toward understanding IRF1’s complex biological roles and utilizing that knowledge to create clinically useful treatments. Proteolysis Targeting Chimeras (PROTACs) provide potential means to achieve selective degradation of IRF1 protein through targeted recruitment of an E3 ubiquitin ligase via a bifunctional molecule, thereby facilitating IRF1’s ubiquitination and subsequent proteasomal degradation. The goal of producing IRF1-specific PROTACs is not yet a reality, but developments in the fields related to proteasomal degradation have aided in creating IRF1-specific PROTACs through understanding factors that affect IRF1 susceptibility to proteolytic degradation. For example, proteasome inhibitors (i.e., bortezomib) increase IRF1 protein stability, which ultimately leads to the upregulation of downstream gene products such as PD-L1 and HLA class I that cause tumors to be more immunogenic (130). The identification of E3 ligases such as TRIM21, and their role in promoting K48-linked ubiquitination of IRF1, provides a mechanistic rationale for the design of degraders for the treatment of sepsis-related injury (59).

CRISPR (Cas9) and CRISPRa have many use cases including protein manipulation and potential for regulating IRF1 transcriptionally, at a genome-wide scale. CRISPR genome-wide screens indicate that IRF1 is a central node in multiple pathways, such as intrinsic resistance to CAR-T cells in the tumor microenvironment (TME) (131) and Regulation of Galectin-9 in T-Cell Acute Lymphoblastic Leukemia (T-ALL) (132). Respectively. This helps to define the role of CRISPRa in enhancing IRF1-mediated anti-tumor immune responses, and CRISPRi in inhibiting IRF1 in hyper-inflammatory states, e.g. septic cardiomyopathy and osteoarthritis (111, 133). Compared to siRNA-mediated knockdowns, CRISPR-based systems possess enhanced specificity and durability (121). In addition, targeting IRF1-associated lncRNAs (e.g., CARINH, IRF1-AS1) with CRISPR represents an indirect but effective means through which to modulate the activity of IRF1 during the context of viral infection and inflammatory bowel disease (48, 55).

A potentially safer therapeutic strategy involves targeting the upstream activators or downstream effectors of IRF1 to achieve more tailored immune modulation. In tumors with JAK1/2 mutations that fail to induce IRF1, proteasome inhibitors or SOX10-IRF4 axis modulators can restore IRF1-driven immunogenicity in a JAK-independent manner (109, 130). Epigenetic modulators, such as HDAC inhibitors, can also activate the SOX10-IRF1 axis to upregulate HLA class I and PD-L1 in sarcomas and melanomas (110). Conversely, in osteoarthritis, inhibiting the recruitment of GCN5 or SETD2 could attenuate IRF1-mediated pro-inflammatory gene expression without affecting overall IRF1 levels (134). Similarly, targeting the regulatory subunit of casein kinase 2 (CSNK2B) may modulate IRF1 DNA-binding affinity to fine-tune antiviral signaling (56). On the effector side, blocking specific downstream molecules like CXCL10, PD-L1 (111, 112)., or CD47 (116)—or inhibiting the IRF1-driven pyroptotic (NLRP3/Caspase-1) (111, 112) and ferroptotic (ALOX15B/GPX4) pathways (135, 136)—represents a clinically viable approach to achieve therapeutic effects with fewer systemic adverse outcomes than global IRF1 knockout.

### 9.3 Exploring the role of IRF1 in novel macrophage subtypes and trained immunity

Modern macrophage biology is moving beyond the binary M1/M2 classification to explore IRF1’s role in specialized subtypes and trained immunity. IRF1 has been identified as a critical regulator of lipid-associated macrophages in atherosclerosis; for instance, the master regulator SUB1 (PC4) promotes atherosclerosis by activating *Irf1* transcription, while its myeloid-specific knockout enhances pro-resolving M2-like polarization and cholesterol efflux (34). Similarly, in the fibrotic niches of the liver, IRF1 governs the inflammatory macrophage genome and sensitivity to ferroptosis (53). Furthermore, the transcription factor Zhx2 cooperates with NF-κB p65 to activate the *Irf1* promoter, a process essential for dictating macrophage subtype specification and anti-tumor responses (36).

IRF1 is also a central player in “trained immunity,” the process by which innate immune cells develop long-term functional memory through epigenetic reprogramming. IRF1 collaborates with factors like ISGF3 and GAF to open inaccessible chromatin and establish *de novo* enhancers that underpin this memory (33). When HSPCs are infected with Mycobacterium avium, they undergo conserved epigenetic changes, such as the presence of elevated H3K4me3 marks and increased accessibility of chromatin at the Irf1 locus, as they differentiate into both monocytes and macrophages (90). When IRF1 is knocked out, the enhanced mitochondrial respiration and bactericidal potential seen in trained cells is compromised, suggesting that IRF1 is a crucial target for trained immunity at the epigenetic level (90). IRF1 is not only a rapid activator in the viral context but also a critical architect of enduring epigenetic alterations that encode the memory of macrophage function and of responses to environmental signals (129).

## 10 Conclusion

The transcription factor IRF1 acts as a context-dependent master regulatory hub governing macrophage polarization. Elevated IRF1 activity initiates transcriptional programs for pro-inflammatory M1-like macrophages and confers host protection against pathogens and tumors, whereas reduced IRF1 abundance de-represses anti-inflammatory M2-like transcriptional signatures. IRF1 activity is fine-tuned through multilayered regulatory circuits operating at transcriptional, epigenetic, post-transcriptional and post-translational levels, and it engages extensive crosstalk with core signaling axes including STAT1 and NF-κB. Beyond directly orchestrating inflammatory gene expression, IRF1 remodels immunometabolic circuits and rewires chromatin accessibility as a pioneer transcription factor. Importantly, IRF1 does not exert simple binary regulatory effects. Under specific pathological microenvironments, sustained high IRF1 activity can paradoxically promote M2-like macrophage phenotypes. Dysregulated or chronically hyper-activated IRF1 drives autoimmunity and excessive inflammatory tissue damage. Taken together, the biological outputs of IRF1 are strongly dictated by cell type, local tissue microenvironment and upstream molecular cues.

Further advances combining single-cell sequencing, multi-omics profiling and well-defined myeloid-specific *in-vivo* knockout models will deepen our mechanistic understanding of IRF1-centered macrophage regulatory networks. Multiple critical unresolved questions remain, including the cell-autonomous functions of IRF1 in restraining or permitting M2-like programming and the contribution of IRF1-driven epigenetic remodeling to trained immunity. Translational targeting of IRF1 still faces considerable practical obstacles. Global activation or inhibition of IRF1 may trigger undesirable systemic adverse effects given its essential physiological role in host defense. Future therapeutic strategies should pursue spatiotemporally precise and cell-type-selective modulation of IRF1. Innovations in nanoparticle-based delivery, selective small-molecule modulators and gene-editing tools will help achieve this goal and unlock new opportunities for treating infectious diseases, malignancies and autoimmune inflammatory disorders.
